# Radiomic features and multilayer perceptron network classifier: a robust MRI classification strategy for distinguishing glioblastoma from primary central nervous system lymphoma

**DOI:** 10.1038/s41598-019-42276-w

**Published:** 2019-04-05

**Authors:** Jihye Yun, Ji Eun Park, Hyunna Lee, Sungwon Ham, Namkug Kim, Ho Sung Kim

**Affiliations:** 1 Department of Convergence Medicine, University of Ulsan College of Medicine, Asan Medical Center, 88 Olympic-Ro 43-Gil Songpa-Gu, Seoul, 05505 Korea; 2 Department of Radiology and Research Institute of Radiology, University of Ulsan College of Medicine, 88 Olympic-Ro 43-Gil Songpa-Gu, Seoul, 05505 Korea; 3 Health Innovation Big Data Center, Asan Institute for Life Science, 88 Olympic-Ro 43-Gil Songpa-Gu, Seoul, 05505 Korea

## Abstract

We aimed to establish a high-performing and robust classification strategy, using magnetic resonance imaging (MRI), along with combinations of feature extraction and selection in human and machine learning using radiomics or deep features by employing a small dataset. Using diffusion and contrast-enhanced T1-weighted MR images obtained from patients with glioblastomas and primary central nervous system lymphomas, classification task was assigned to a combination of radiomic features and (1) supervised machine learning after feature selection or (2) multilayer perceptron (MLP) network; or MR image input without radiomic feature extraction to (3) two neuro-radiologists or (4) an end-to-end convolutional neural network (CNN). The results showed similar high performance in generalized linear model (GLM) classifier and MLP using radiomics features in the internal validation set, but MLP network remained robust in the external validation set obtained using different MRI protocols. CNN showed the lowest performance in both validation sets. Our results reveal that a combination of radiomic features and MLP network classifier serves a high-performing and generalizable model for classification task for a small dataset with heterogeneous MRI protocols.

## Introduction

Distinguishing primary central nervous system lymphoma (PCNSL) from glioblastoma is an important task in neuro-oncology because treatment options are vastly different for the two diseases^1,2^. In this regard, the development of an imaging-based classification system would be beneficial, which will provide the desired improvements in diagnostic accuracy, by utilizing imaging-based features, such as histograms, texture features, and transformed features. This radiomic approach converts sparse magnetic resonance imaging (MRI) data into big data by generating high-dimensional imaging phenotypes from the given imaging data in a voxel-wise model^3,4^. Combined with machine learning classifiers, the radiomics approach has improved diagnostic performance for histologic grading, predicting molecular markers, and improving diagnosis using MRI data^5–9^.

On the other hand, recently introduced deep neural networks, especially convolutional neural networks (CNNs), have improved classification performance in various medical applications, including the diagnosis of tuberculosis, diabetic retinopathy, and skin cancers using chest X-ray scans^10^, fundal photographs^11^, and digital images^12^, respectively. Deep features can be extracted from the pre-trained CNN^13^, thereby allowing for the modeling of high-level abstractions from data and automatic feature discovery without using a pre-defined feature selection method. Compared to deep features obtained from CNN, traditional radiomic features are handcrafted and pre-designed^13^ and reguire further steps for selection^14^.

Nevertheless, a shortcoming of deep learning is that a large amount of data is needed to minimize overfitting and improve learning^15^. Big data may be feasible with X-rays or photographs, but it is difficult to achieve with brain tumor MRI data. The low incidence figures for PCNSL (0.47 cases per 100,000 person-years^16^) and glioblastoma (2.2 to 3.7 cases per 100,000 person-years^17^) make it practically challenging to obtain the hundreds to thousands of MRI images required for deep learning. Thus, a better classification strategy is needed for deep learning involving MRI data, with different combinations of data input, feature extraction, and classifiers.

In view of the above issues, we aimed to establish a high-performing classification strategy using a small MRI dataset. The classification task can be greatly affected by different feature extraction (handcrafted vs. deep features) and classification (support vector machine, generalized linear model, random forest, multilayer perceptron (MLP) network, and CNN) methods, but there are few published studies comparing classification strategies that vary based on these methods. Also, classification system performance for MRI data may vary by imaging protocol because machine learning is fine-tuned with the training dataset. The purpose of this study, therefore, was to find a high-performing and robust classification strategy for MRI using different combinations of feature extraction, feature selection, and classifiers from human readers, supervised learning, and deep learning with regard to a neuro-oncologic diagnosis task involving a small dataset.

## Results

The overall patient demographics and image protocols of the training and validation sets are listed in Table 1. Between training and internal validation and between training and external validation set, there was no significant difference in proportion of glioblastoma and PCNSL (chi-square test, *P* = 0.948 and *P* = 0.400, respectively), proportion of female patients (chi-square test, *P* = 0.151 and *P* = 0.103, respectively), and age (independent samples *t*-test, *P* = 0.651 and *P* = 0.323, respectively).

**Table 1:** Demographics of the Patients and Protocols in the Image Datasets.

Group	Dataset origin	No. of glioblastoma	No. of PCNSL	Age (y)	Female (%)	MR	TR/TE (ms), CE-T1WI	Slice thickness (mm), CE-T1WI	TR/TE (ms), DWI	Slice thickness (mm), DWI
Training set	AMC	73	50	63.0 ± 11.7	35.9	3 T	9.0–10.1/4.4–4.8	0.5	3000–4000/56–61.7	4 mm
Internal validation set	AMC	18	12	58.8 ± 13.0	50.0	3 T	9.0–10.1/4.4–4.8	0.5	3000–4000/56–61.7	4 mm
External validation set	SMC	28	14	56.3 ± 10.6	50.0	3 T	8.6–10.4/3.5–4.7	1	6900–12,000/55–81	3–5 mm

Abbreviations: Data are expressed as mean ± standard deviation for age and range for image protocols. PCNSL = primary central nervous system lymphoma, TR = repetition time, TE = echo time, CE-T1WI = contrast-enhanced T1 weighted imaging, DWI = diffusion weighted imaging, AMC = Asan Medical Center, Seoul, Korea, SMC = Samsung Medical Center, Seoul, Korea.

### Optimizing Metric 1, 2, and 4 using Data Training and Internal Validation

**Figure 1:**
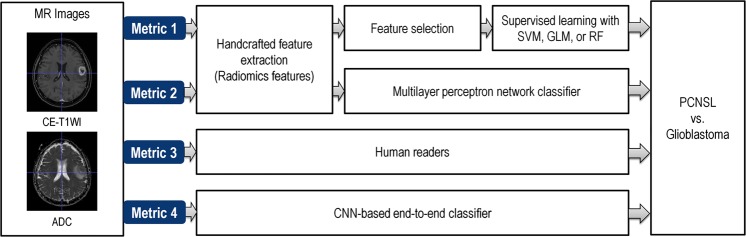
Study design. Metric 1: extraction of radiomic features, followed by machine learning of support vector machine (SVM), generalized linear model (GLM), and random forest (RF); Metric 2: extraction of radiomic features, followed by deep learning-based classification of multilayer perceptron network; Metric 3: assessment by 2 neuroradiologists; Metric 4: end-to-end classifier using convolutional neural network (CNN) without feature extraction.

The study design is demonstrated in Fig. 1. Metric 1 is machine learning classifier for radiomics features; metric 2 is deep learning-based classification of multilayer perceptron network for radiomics features; metric 3 is assessment by two neuroradiologists; metric 4 is convolutional neural network (CNN) without feature extraction.

**Figure 2:**
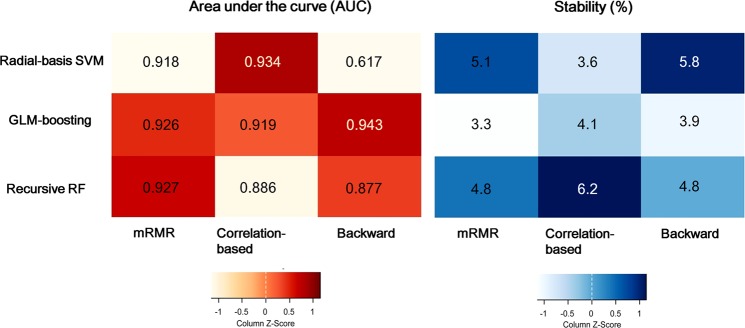
Optimization of metric 1 in the training set using contrast-enhanced T1-weighted (CE-T1WI) and ADC images. (**A**) Heatmap depicting the diagnostic performance (area-under-the receiver operating characteristics curve, AUCs) of 3 feature selection (in rows) and 3 classification (in columns) methods in the training set. Color scale: expressed from orange (AUC, 0.60) to red (AUC, 1.00). (**B**) Stability of the AUCs using 10-fold cross-validation in the training set. Color scale: expressed from sky blue (stability, 3%) to dark blue (stability, 6.5%).

Figure 2 demonstrates the area under the receiver operating characteristic curves (AUCs) and stability in metric 1 using different combinations of machine learning algorithms, feature selection, and classifiers by employing radiomic features from both contrast-enhanced T1-weighted (CE-T1W) and apparent diffusion coefficient (ADC) images. Among the nine different combinations, the combination of backward feature elimination and generalized linear model (GLM) boosting showed the best diagnostic performance in the training set, which had a mean AUC of 0.945 (95% CI: 0.916–1); sensitivity, 96.3%; specificity, 92.3%; and accuracy, 94.3%. The stability of the best combination was 3.9%. The results using nine different combinations of feature selection and classification methods are shown in the Supplementary Table 3. When metric 1 was tested with each CE-T1W or ADC image, the mean AUCs ranged from 0.834 to 0.924 among the CE-T1W images, and the best performance was achieved with correlation-based feature selection (CFS) and radial-basis support vector machine (SVM). For ADC maps, the mean AUCs ranged from 0.827 to 0.910, and the best performance was obtained with CFS and regularized RF. The selected features from the best combination are shown in the Supplementary Table 4. The selected algorithm in metric 1 was further applied to internal and external validations.

Metric 2 was optimized with 100-10 network architecture using both CE-T1W and ADC images, with an AUC of 0.991 (95% CI: 0.987–0.994) in the internal validation set. Its performance decreased with deeper networks, especially when using both CE-T1W and ADC images and when using ADC images only. Table 2 shows the optimization of classification the using MLP network (metric 2) and CNN (metric 4) by comparing the diagnostic performance in the internal validation set.

**Table 2:** Optimization of the best classifier in multilayer perceptron network (metric 2) and CNN (metric 4) by comparing diagnostic performance in the internal validation set.

Imaging data	CE-T1WI + ADC	CE-T1WI	ADC
MLP network	AUC (95% CI)		
100-10	0.991 (0.987–0.994)	0.965 (0.959–0.972)	0.969 (0.960–0.978)
500-100-10	0.990 (0.987–0.993)	0.965 (0.960–0.971)	0.968 (0.956–0.979)
500-100-50-10	0.989 (0.985–0.993)	0.956 (0.949–0.962)	0.964 (0.953–0.975)
500-250-100-50-10	0.988 (0.982–0.995)	0.968 (0.964–0.982)	0.965 (0.952–0.977)
750-500-250-100-50-10	0.986 (0.978–0.993)	0.971 (0.967–0.975)	0.960 (0.951–0.969)
CNN	Accuracy (Sensitivity/Specificity)		
Inception v-3	80.0 (50.0/100)	76.7 (41.6/100)	86.7 (83.3/88.9)

Abbreviations: MLP = multilayer perceptron, CNN = convolutional neural network, CE-T1WI = contrast-enhanced T1 weighted imaging, DWI = diffusion weighted imaging, AUC = area under the receiver operating characteristic curve.

**Figure 3:**
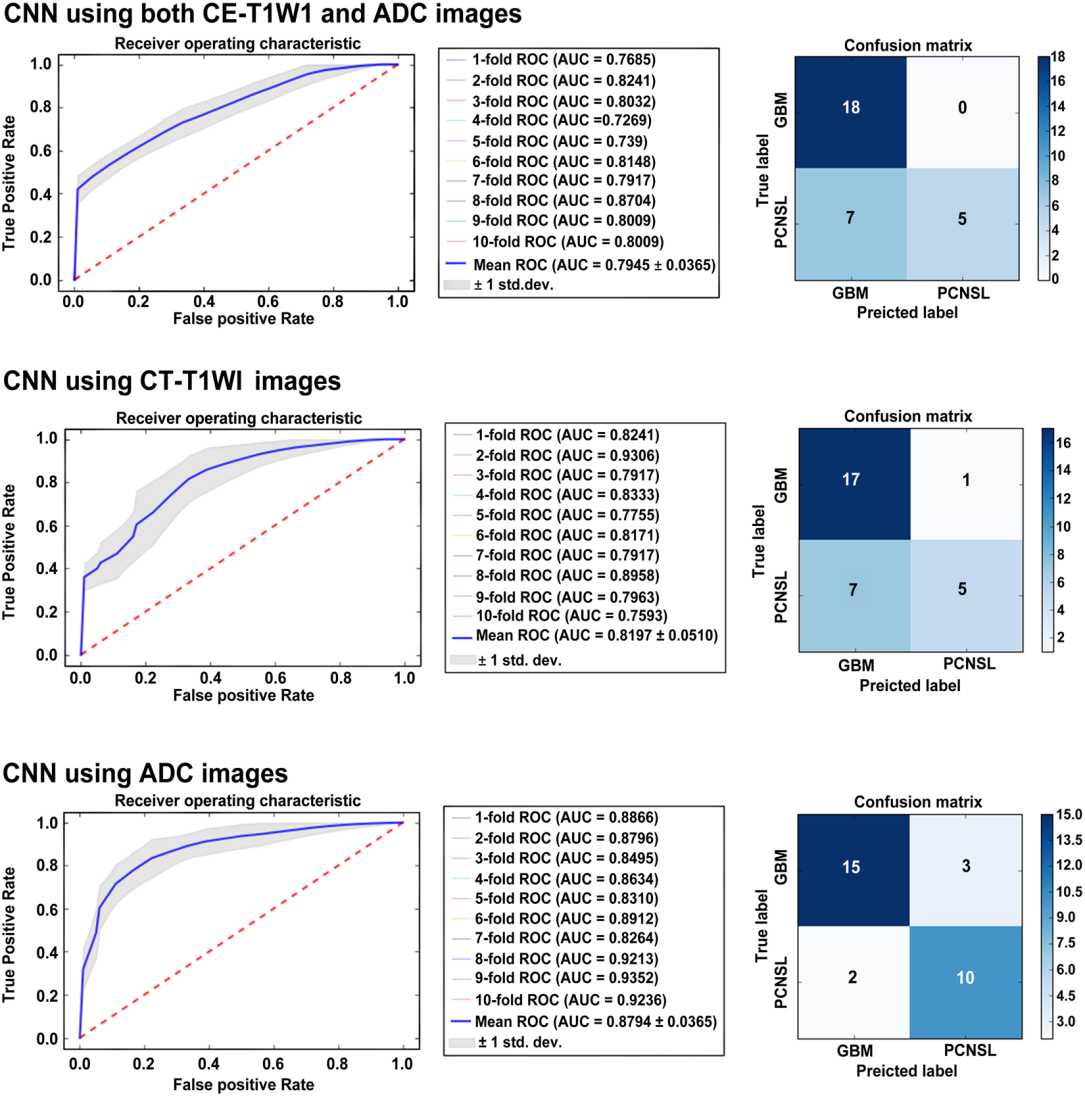
The difference of receiver operating characteristic curve according to image sets in the convolutional neural network (CNN) classifier (metric 4). The CNN showed the best performance with apparent diffusion coefficient (ADC) images, compared with the performance achieved when a combination of contrast-enhanced T1-weighted (CE-T1WI) and ADC images were used or when CE-T1WI images alone were used.

In metric 4, the best performing model, diagnostic performance was higher on using ADC images alone (AUC, 0.879; 95% CI: 0.856–0.902) as compared with that achieved on using CE-T1W images (AUC, 0.819; 95% CI: 0.788–0.851) and both image sets (AUC, 0.794; 95% CI: 0.772–0.817). Figure 3 demonstrates the differences of metric 4 in the internal validation set.

The simple logistic regression classifier provided a diagnostic performance of AUC 0.939 when both CE-T1W and ADC images were used in the internal validation set, which was lower than that achieved using the MLP network. The diagnostic performance achieved by using only CE-T1W or ADC images was AUC 0.867 and AUC 0.875, respectively.

### Diagnostic performance in the external validation set

Human readers did not need training from data, and the diagnostic performance was directly calculated in the validation sets. In the internal validation, human readers showed AUCs of 0.833 and 0.875 (95% CI: 0.653–0.940 and 0.703–0.967), with sensitivities of 83.3% and 75.0%, specificities of 83.3% and 100%, and accuracies of 83.3% and 90.0%, respectively. In the external validation set, human readers showed AUCs of 0.913 and 0.930 (95% CI: 0.808–0.971 and 0.831–0.981), with sensitivities of 86.2% and 89.7%, specificities of 96.4%, and accuracies of 91.2 and 93.0%.

**Figure 4:**
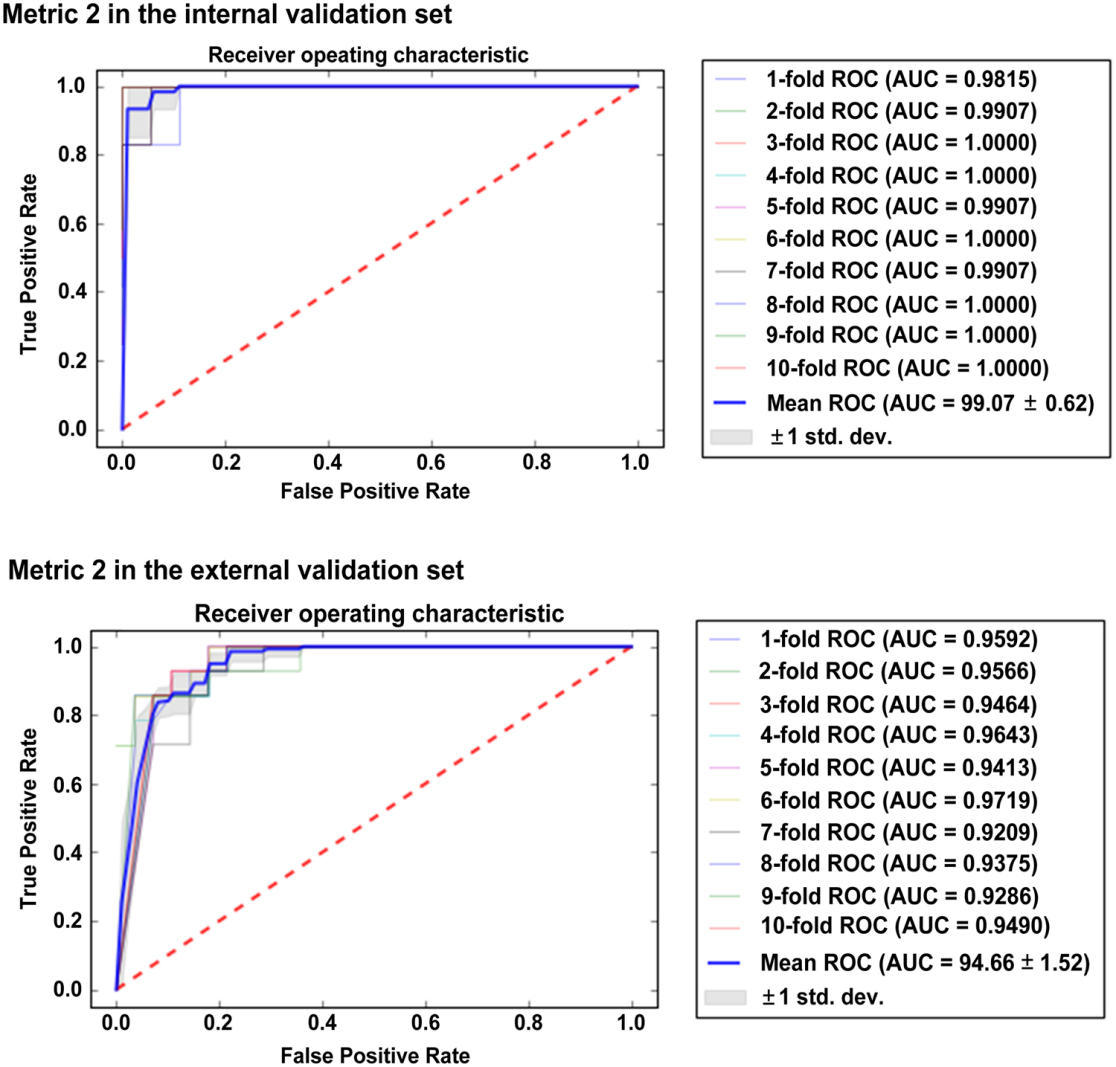
The diagnostic performance of metric 2 in the internal and external validation sets. The area under the receiver operating characteristic curve (AUC) remained robust in the external validation set.

Table 3 summarizes the diagnostic performance in the training and validation sets. The decrease in the diagnostic performance of metric 4 was the most substantial, from an AUC of 0.879 (95% CI: 0.856–0.902) in the internal validation to 0.486 (95% CI: 0.468–0.503) in the external validation set. The diagnostic performance of metric 1 also reduced in the external validation, from an AUC of 0.931 (95% CI: 0.891–0.962) in the internal validation to an AUC of 0.811 (95% CI: 0.795–0.835) in the external validation. Metric 2 remained robust in the external validation, with an AUC of 0.947 (95% CI: 0.937–0.956), a sensitivity of 92.9%, a specificity of 82.1%, and an accuracy of 85.7%. The diagnostic performance of metric 2 was the highest in the external validation, followed by that of human readers (metric 3). Figure 4 shows the AUCs of metric 2 in the validation sets.

**Table 3:** Validation Datasets Showing the Area Under the Curve, Sensitivity, and Specificity of the Classification Metrics to Distinguish Primary Central Nervous System Lymphoma and Glioblastoma, With Reference to a Histopathology Finding.

Metric		Images	Training set			Internal validation set			External validation set		
			AUC (95% CI)	Sens (%)	Spec (%)	AUC (95% CI)	Sens (%)	Spec (%)	AUC (95% CI)	Sens (%)	Spec (%)
1	Backward feature elimination with GLM boosting classifier	Radiomics features	0.943 (0.927–0.978)	96.3	92.3	0.931 (0.914–0.941)	98.8	92.3	0.811 (0.795–0.835)	85.5	78.9
2	MLP network classifier (100-10)	Radiomics features	0.994 (0.994–0.995)	100	100	0.991 (0.987–0.994)	100	100	0.947 (0.937–0.956)	92.9	82.1
3	Human readers	Images	0.825–0.908 (0.755–0.949)	69.4–83.9	95.6–97.8	0.833–0.875 (0.653–0.940)	75.0–83.3	83.3–100	0.913–0.932 (0.808–0.981)	86.2–89.7	96.4–96.4
4	CNN based end-to-end classifier	Images	0.973 (0.966–0.980)	100	94.51	0.879 (0.856–0.902)	83.3	83.3	0.486 (0.468–0.503)	100	35.7

Abbreviations: GLM = Generalized linear model, MLP = multilayer perceptron, CNN = convolutional neural network, AUC = area under the receiver operating characteristic curve, Sens = Sensitivity, Spec = Specificity.

### Post-hoc analysis for the impact of CNN-based deep features on radiomics features

Comparison between the diagnostic performance of radiomic features and that of CNN-based deep features combined with radiomic features is shown in Supplementary Table S5. In the internal validation, radiomic features involving the use of both CE-T1WI and ADC images yielded an AUC of 0.9907, but the AUC dropped to 0.4722 when deep features were added. Similar findings were observed in the external validation, in which radiomic features yielded an AUC of 0.9260 which decreased to 0.7895 when deep features were added.

## Discussion

In this MRI-based image classification task, a deep learning-based MLP network classifier with radiomic features showed the highest performance in differentiating PCNSL from glioblastoma. Combination of backward feature elimination and GLM classifier using radiomic features showed a comparable performance in the internal validation set, but the performance dropped significantly in the external validation set with different MRI protocols. Human readers showed slightly lower diagnostic performance than machine or deep learning, but performance remained robust across different datasets. The CNN trained with 123 MR image sets without feature extraction showed the worst performance in both validation sets. Our results demonstrated that a MLP network classifier, combined with radiomic features, is a useful tool for MRI data, with improved diagnostic performance and avoidance of overfitting across the different protocols.

The major difference between metrics 1, 2, and 4 was the feature extraction strategy—whether features were extracted manually (radiomic features in metrics 1 and 2) or automatically learned by the system (metric 4). A CNN is an end-to-end deep learning algorithm, where the cascades of convolution-pooling layers mimic the extraction of visual features and the fully connected layers are incorporated to integrate all the feature responses and provide the final results^15^. Thus, deep learning extracts high-level features directly from the data. However, CNNs are intrinsically data hungry and susceptible to overfitting^18^; they require many data samples for model training because millions of weight parameters are needed to be estimated within the network. A previous study has suggested that at least 100 cases per class are needed to provide a reasonable outcome^19^. The poor performance of CNN in our study is in keeping with the limitations associated with extracting deep features using a small dataset, even with data augmentation and use of a pre-trained network. The results of our study emphasize that MRI data are vastly different from 2D single-plane imaging data of digital photos, which are commonly used in CNN image-based classification.

On the other hand, radiomics enables the conversion of MRI images into high-dimensional feature spaces that allow an improved performance in PCNSL and glioblastoma diagnoses. Three aspects are to be established in radiomics modeling: feature selection, modeling, and validation^20^. An effective feature selection is a crucial step^20^ because radiomics features are multiple collinear and correlated predictors that could produce unstable estimates and might overfit predictions^14^. Additionally, performance is preferably assessed with a true validation set, both temporally and geographically, and problems in the model fitting stage will reflect as poor performance in the validation stage. In our study, combination of backward elimination and GLM classifier (metric 1) and MLP network (metric 3) showed similarly high performances in internal validation sets. However, the metric 1 showed reduced diagnostic performance in the external validation, which can be explained by overfitting the training MRI data, owing to the use of an overly simplified feature selection method.

Meanwhile, the MLP network (metric 2) applied to radiomic features provided the best performance and remained robust in both validating sets across different MR imaging protocols. The MLP network models the computational units of multiple layers by imitating signal transmission, and the layers of deep neural architecture overcome the limitation of local minimum optimization^19^. Also, an ensemble model that we employed using 10-fold cross-validation methods enabled us to overcome overfitting^21–23^ and to classify more complex and nonlinear relationships by adding more hidden layers to the network architecture between the input and output layers. The rationale behind application of MLP was that it has hidden layers with the capability of producing a higher level and more abstracted feature selection algorithm. MLP-based feature selection may overcome overfitting of radiomics features trained and selected from the same institute, which was shown with metric 1, and may be prone to more efficiently adopt and select significant radiomics features from different MRI acquisitions in the external validation set. This approach is different from that for a CNN, in which a model learns from data with deep architecture in an unsupervised manner and generates features from raw data. Though CNNs avoid complex feature engineering or delicate feature handcrafting, as shown in our study, they may not efficiently extract features from small MRI datasets and classify independent data with heterogeneous MRI acquisition protocols. Based on our results, we recommend the combination of radiomic features and MLP network classification as a suitable analysis strategy when there are limited MRI data.

A previous study has showed that extraction of deep learning information from MR images will improve diagnostic efficacy than that associated with the radiomics model^24^, but this was tested using the same imaging protocol. Based on our results, compared to the radiomic features alone, addition of CNN-based deep features does not improve diagnostic performance. This is probably because the extracted features from radiomic analysis and CNN algorithm have different dimensions, and the concatenating features did not improve model performance.

Of note, human performance showed lower diagnostic performance in the internal validation but emerged as the best performer (metric 2) in the external validation. In learning, humans take general principles and apply theory to new data^18^, while machines learn directly from given data. The neuroradiologists in our study did not need to learn from the training data, but they applied their knowledge-based rules in both validation sets. A discrepancy between the two validation sets may result from the inclusion of atypical PCNSL cases^25^, showing necrosis and hemorrhage in the internal validation set, which resulted in reduced diagnostic performance of human readers. Meanwhile, quantitative imaging features from radiomic feature extraction improved classification in both validation sets.

The training and validation data used here was used in our previous work^26^ comparing several ML models. This study differs from previous work by first applying MLP network classifier and CNN based end-to-end classifier to radiomics in order to alleviate overfitting issue from the overly simplified feature selection method. Although previous study showed high diagnostic performance of AUC 0.984 in the internal and AUC 0.944 in the external validation set, the study utilized larger number of radiomics features (n = 1618) compared to the current study (n = 936). Also, applying multiple different ML combinations of feature selection and classifiers is extensive work and may not be suitable to explain complex and nonlinear relationships. In this study, the MLP network classifier showed robust across different protocols and centers, showed diagnostic performance of AUC 0.991 in the internal validation set and AUC 0.947 in the external validation set. This is further supported that the sensitivity and specificity of the external validation set (85.7% and 75.0%, respectively) was lower in the previous study than in the current study (92.9% and 82.1%, respectively).

Our study has several limitations. First, it is conducted with only a small amount of data, especially in the validation set. Second, though we used a modified network from pre-trained Inception-v3, CNN performance did not reach to metric 1 or metric 2. This may result from Inception-v3 being optimized for 2D images, and not directly from the MRI data. More suitable and optimized networks can further improve CNN model performance. Third, combination of CNN and radiomics features were based on lower dimension (2D slice), and this may have lowered the diagnostic performance of combined approach compared to using radiomics features only. In future work, feature extraction using 3D CNN will be more desirable, which can simultaneously extract both spatial and multimodal information of multimodal MRI data^27^. Fourth, radiomic feature extraction is inherently time-consuming and subject to variations in segmentation and imaging sequences, and the best performer of radiomic features and MLP network classifiers in this study may not lead us to identify a clinically useful tool. Nevertheless, a comparison of the performance between human learning and various machine learning may give insights on how learning systems can be improved. Finally, atypical PCNSL cases may consist of different HLA–typed PCNSL; correlation of molecular markers to imaging phenotypes will improve the biologic relevance of the current research.

In conclusion, a combination of radiomic features and MLP network classifier serves as a high-performing and reliable model for a classification task and is superior to support vector machine, generalized linear model, random forest, and CNN for small datasets with heterogeneous MRI protocols.

## Methods

### Image datasets

We collected MR image sets from patients who were pathologically confirmed to have PCNSL or glioblastoma by surgical resection or biopsy. From the electronic database of our institution, 153 contrast-enhanced T1-weighted (CE-T1W) and diffusion-weighted (DW) MR image sets were collected for each patient. These types of imaging data were selected because both are sequences in standardized brain tumor protocols for clinical trials^28^ and both are frequently used as diagnostic tools in conventional^1,29^ and advanced^30,31^ imaging studies to distinguish two entities. Ninety-one patients had glioblastoma, and 62 patients had PCNSL. The imaging data were randomized into a training set (123 MR image sets, 50 PCNSLs, and 73 glioblastomas) and an internal validation set (30 MR image sets, 12 PCNSLs, and 18 glioblastomas). Only the training set was used in model construction.

An external validation set with 42 MR image sets (14 PCNSLs and 28 glioblastomas) was also collected, from another tertiary hospital, to test the effect of different acquisition protocols on model performance. The institutional review board of Asan Medical Center approved this retrospective study, and the requirement for informed consent was waived for both centers. The imaging data were de-identified in accordance with the Health Insurance Portability and Accountability Act privacy rule.

### MR Image Preprocessing

MR images were acquired using 3T scanners at both centers. CE-T1W and DW MR images were collected for image analysis. In our hospital, the CE-T1W images were obtained at a high-resolution three-dimensional (3D) volume, using a gradient-echo T1-weighted (T1W) sequence with the following parameters: repetition time (TR)/echo time (TE), 9.8/4.6 ms; flip angle, 10°; field of view (FOV), 256 mm; matrix, 512 × 512; and slice thickness, 1 mm with no gap. DW image parameters were as follows: TR/TE, 3000/56 ms; diffusion gradient encoding, b = 0, 1000 s/mm^2^; FOV, 25 cm; matrix, 256 × 256; and slice thickness/gap, 5 mm/2 mm.

For the external validation set, CE-T1W images were obtained using a gradient-echo T1W sequence: TR/TE, 7.6/3.7 ms; flip angle, 10°; FOV, 24 mm; matrix, 512 × 512; and slice thickness, 1.2 mm with no gap. DW image parameters were as follows: TR/TE, 3000/46.2–82.2 ms; diffusion gradient encoding, b = 0, 1000 s/mm^2^; FOV, 24 cm; matrix, 128 × 128; and slice thickness/gap, 5/2 mm. The detailed parameters for imaging data are given in Table 1.

### Preprocessing of MR imaging data

Signal intensity normalization was applied for CE-T1W images using the ANTsR^32^ and WhiteStripe packages^33,34^ in R version 3.3.3 (R Core Team, Vienna, Austria). For DW images, the ADC map was calculated using a two-point estimate of signal decay. ADC outliers ± 3 standard deviations from the mean were removed. Then, CE-T1W and ADC images were co-registered using SPM software (www.fil.ion.ucl.ac.uk/spm/), using affine transformation with normalized mutual information as a cost function^35^, with 12 degrees of freedom and tri-linear interpolation. CE-T1W and ADC images were resampled into a uniform voxel size of 1 × 1 × 1 mm as the input data. These images became an input for metric 3 and 4.

### Tumor segmentation and radiomic feature extraction

3D regions-of-interest (ROIs) for contrast-enhanced portions were semi-automatically identified by a neuroradiologist (J.Y.K., with 2 years of experience) on CE-T1W images using a segmentation threshold and a region-growing algorithm in the Medical Imaging Interaction Toolkit (MITK) software platform (www.mitk.org, German Cancer Research Center, Heidelberg, Germany)^32^. An experienced neuroradiologist (H.S.K., with 18 years of experience) manually edited the ROIs.

From the segmented mask, 936 total radiomic features were extracted using Matlab R2015a (MathWorks Inc., Natick, MA). The features included 17 first-order features, 87 texture features, and 832 wavelet features. The first-order features were derived from the intensity histograms using first-order statistics, including intensity range, energy, entropy, kurtosis, maximum, mean, median, uniformity, and variance. Texture features were obtained from a gray-level co-occurrence matrix (GLCM) and a gray-level run-length matrix (GLRLM)^36^ using the segmented mask in 13 directions of a 3D space. For GLCM analyses, texture features were computed for varying distances of 1, 2, and 3 voxels in 13 directions. Then, wavelet transformation was applied with a single-level directional discrete wavelet transformation of a high-pass and a low-pass filter^37^. In total, eight wavelet-decomposition images were generated from each MRI sequence input: LLL, HLL, LHL, HHL, LLH, HLH, LHH, HHH images, where ‘L’ means ‘low-pass filter’ and ‘H’ means ‘high-pass filter’. The first-order features and texture features were then applied to the wavelet-transformed images (17 first-order features +87 texture features) multiplied by 8 images, yielding 832 wavelet features. All radiomic features were z transformed before applying classification metrics. The details of the radiomic feature extraction are described in the Supplementary Materials. The radiomic features become inputs for metrics 1 and 2.

### Classification metrics

Four different classification metrics were applied using radiomic features or preprocessed MRI data. Metrics 1 and 2 used radiomic features, and feature selection and classification were optimized with SVM, GLM, or random forest (metric 1) or multilayer perceptron (MLP) network (metric 2). Metrics 3 and 4 used preprocessed MR images, and classification was performed by human readers (metric 3) or an end-to-end CNN (metric 4).

### Metric 1

As many extracted features may be noise, or highly correlated with each other, feature reduction or selection is required to increase the prediction accuracy and minimize computational costs^38^. Supervised learning algorithms were implemented for feature selection and classification methods using R. Three feature selection methods were used in the analysis: minimum redundancy maximum relevance (mRMR), correlation-based feature selection (CFS), and backward elimination. Three different classifiers were used: radial-basis support vector machine^39^, the boosted generalized linear mixed model^40^, and the regularized random forest (Supplementary Materials). The algorithms were selected based on their high performance and readiness for application^9,41^. Using three feature selection methods and three classifiers, nine different models were computed and compared to determine the best combination for diagnosing PCNSL from glioblastoma in the training set. The models were developed separately for each of the CE-T1W and ADC images as well as for the combined CE-T1W and ADC images. Classifiers were trained using 10-repeat iterations and 10-fold cross-validations of the training set, which allows repetition of experiments for each model up to 100 times. The classification performance was evaluated in training set as well as in both validation sets.

### Metric 2

A MLP network allows for modeling of high-level abstractions from data and automatic discovery of features. In total, four MLP networks were constructed including 100-10, 500-100-10, 500-100-50-10, and 500-250-100-50-20. Before training the constructed MLP networks, all datasets were standardized to make training faster and minimize the probability of getting stuck in local optima. All four MLP networks were trained by minimizing training error (namely, the cross-entropy error between the inferred label and the true label) by stochastic gradient descent (SGD), and the hyper-parameters were batch size 32, momentum 0.25, and learning rate 10^−3^ without weight decay. Since glioblastoma had a higher number of data points than PCNSL and this class imbalance can have a significant detrimental effect on both convergence during the training phase and generalization of a model on the test set, we added class weights (1.467 for PCNSL) to the loss function. Accuracies (including sensitivities and specificities) were evaluated by ensembles of the 10 trained models that were obtained during the 10-fold cross-validation of training sets. Instead of selecting a single classifier, ensemble learning methods train several baseline models and use some rules to combine them to make better decisions^21–23^. As baseline models, we selected 10 trained models obtained via 10-fold cross-validation, and then combined them with majority voting. Further, to measure activation functions and optimization methods of MLP in classification, a simple logistic regression is applied for comparing the classification performance between the validation sets. The cross-validation methods were applied in a manner similar to that applied in the MLP network.

### Metric 3

Two radiologists (one was a senior with 2 years of subspecialty training in neuroradiology and the other was a one-fourth year resident) were recruited to test human performance. MR image sets including CE-T1W and DW images and ADC maps were allocated to a separate workstation after data anonymization and randomization. These image sets were provided to include essential sequences and make proper comparisons with other metrics. The three aforementioned image sets were presented at the same time, and the readers were assigned all data sets at once to prevent learning effect and recall bias. Validation data sets (for 30 internal and 42 external validation sets, respectively) were assigned in a flexible session for 1 week, and readers were asked to rate their level of confidence in their diagnoses using one of the following labels: “definitely PCNSL,” “probably PCNSL,” “probably glioblastoma,” or “definitely glioblastoma.”

### Metric 4

We employed CNN with an end-to-end approach that integrates an automatic feature extraction and a discriminative classifier into one model. To overcome the small number of data points, we employed Inception-v3 of a 2-dimensional (2D) image-based CNN and built bigger datasets by converting 3D MR images to 2D images. The training set of 123 MR image sets (50 PCNSLs and 73 glioblastomas) was converted to 4,714 slices (1,674 PCNSL slices and 3,040 glioblastomas slices). In addition, transfer learning was used, and a pre-trained Inception-v3 model was fine-tuned to perform classification of our MR images, thereby improving the training speed and quality. The Inception-v3 model was pre-trained on the ImageNet (http://www.image-net.org/) dataset comprising 1.2 million color images organized into 1,000 categories. We converted the selected 2D image slices into color images and resized them to Inception-v3 input size (299 × 299 mm). For combining CE-T1W images and ADC maps together, we encoded the CE-T1W images for the red and green channels and ADC map for the blue channel. Because our trained CNN assumed that the tumor regions were segmented, internal and external validations were also performed with the slices overlapping with tumors. Slices for each patient were classified by our trained CNN, and then the final decision was performed by majority voting. In the same manner of the neural net algorithm (metric 2), the accuracy of this end-to-end approach was evaluated by the ensembles of 10 trained models, which were obtained via 10-fold cross-validation of the training sets. Given the training dataset, metric 4 was learned by minimizing the training error (namely, the cross-entropy error) between the inferred label and the true label by SGD. The hyper-parameters were batch size 32, class weight 1:1.791 between glioblastoma and PCNSL, and learning rate 10^−4^ without weight decay. Each baseline model for the ensembles was trained until 600 epochs, and then the optimal models were selected with the best performance on the validation sets. From the trained CNN, 1024 deep features of the last fully connected layer were extracted.

### Assessment of the impact of CNN-based deep features on the radiomic features

In post-hoc analysis, the impact of the deep features to the radiomic features was assessed to observe the impact of raw MRI images as imaging features. The 1024 deep features were extracted from the last fully connected layer of CNN and they concatenated with radiomic features. Random forest was used as the classifier, and the diagnostic performance of a model using radiomic features alone was compared with that of combined radiomics and CNN-based deep features applied with random forest classifier. Since the radiomics features and CNN-based deep features were extracted from the different dimensionality (3D-based radiomics features vs. 2D-CNN-based deep features), two-type features were combined based on lower dimension (2D slice), and diagnostic performances were evaluated by per-patient decision. Grid search was performed to find optimal hyperparameters for random forest classifier using sklearn in Python (using GridSearchCV library).

### Statistical analysis

Differences in patients between training and internal validation set and between training and external validation set were evaluated using independent samples *t*-tests and Chi-square test. From a total of 195 MR image sets, 123 sets were trained for distinguishing PCNSL from glioblastoma for metrics 1, 2, and 4; the performance of each metric was evaluated using 30 MR image sets in the internal validation and 42 sets in the external validation. Each metric was developed and trained in the training set and its diagnostic performance was tested in both the internal and external validation sets. AUCs were used to determine diagnostic performance, with optimal thresholds of the imaging parameters determined by maximizing the sum of the sensitivity and (1 − specificity) (the Youden index) values that were calculated to differentiate between the two entities. Two-by-two contingency tables were created to determine accuracy, sensitivity, and specificity.

In metrics 1 and 2, we further calculated the stability of the subsampled cohort in the training set (size n/10) using relative standard deviation (RSD)^41^. RSD was defined as a percentage as per the following mathematical formula: (standard deviation of AUC/mean of AUC) × 100. RSD was used to determine the robustness and accuracy of a model. *P* values of < 0.05 were considered statistically significant.

## Supplementary information

Supplementary Table

## Data Availability

The datasets generated during and/or analysed during the current study are available from the corresponding author on reasonable request.
