# Supplementary material for: Radiomic features and multilayer perceptron network classifier: a robust MRI classification strategy for distinguishing glioblastoma from primary central nervous system lymphoma
Source: Sci Rep. 2019 Apr 5;9:5746. doi: 10.1038/s41598-019-42276-w (PMC6451024; doi:10.1038/s41598-019-42276-w)
Supplement: Supplementary file 1 — Supplementary Table [file 41598_2019_42276_MOESM1_ESM.docx]

Supplementary Materials

**Radiomic features and multilayer perceptron network classifier: a robust MRI classification strategy for distinguishing glioblastoma from primary central nervous system lymphoma**

Jihye Yun^1^, Ji Eun Park^2^, Hyunna Lee^3^, Sungwon Ham^1^, Namkug Kim^1,2^, and Ho Sung Kim^2^

^1^ Department of Convergence Medicine, University of Ulsan College of Medicine, Asan Medical Center, [88 Olympic-Ro 43-Gil Songpa-Gu, Seoul, South Korea](https://maps.google.com/?q=88+Olympic-Ro+43-Gil+Songpa-Gu,+Seoul,+South+Korea&entry=gmail&source=g), 05505

^2^ Department of Radiology and Research Institute of Radiology, University of Ulsan College of Medicine, Asan Medical Center, Seoul, Korea

^3^ Health Innovation Big Data Center, Asan Institute for Life Science, Asan Medical Center, Seoul, Korea.

Corresponding author: Ji Eun Park, M.D., Ph.D.

Department of Radiology and Research Institute of Radiology, University of Ulsan College of Medicine, Asan Medical Center, 43 Olympic-ro 88, Songpa-Gu, Seoul 05505, Korea.

Phone: 82-2-3010-1505. E-mail: [jieunp@gmail.com](mailto:jieunp@gmail.com)

**S1. Radiomic features**

**S2. Feature selection and classification methods in machine learning**

**S3. Results of metric 1 in each magnetic resonance (MR) imaging dataset**

**S4. Results of adding deep features to radiomics features in random forest classifier**

**S1. Radiomic features**

The imaging features that were calculated were divided into three groups: 17 first-order statistics and 162 texture features. Wavelet transformation was applied to the apparent diffusion coefficient (ADC) and post-contrast T1-weighted (T1W) images, and eight wavelet-decomposition images were generated from each magnetic resonance (MR) image that was input. Then first-order features and texture features were calculated from the eight wavelet decomposition images, which further resulted in 936 wavelet features ([17 + 87] × 8). In total, 936 features (17 first-order statistics, 162 texture features, and 832 wavelet features) were obtained from contrast-enhanced T1-weighted (CE- T1W) and ADC images.

1. First-order statistics

First-order statistics were calculated from the histogram of voxel intensities, which represents the distribution of gray values within an image. Let *P* denote the first-order histogram of a volume of interest V(x,y,z) with isotropic voxel size and *P*(*i)* describe the number of voxels with gray level *i*. The number of gray-level bins set for *P* is represented as Ng. The *i*th probability vector of the first-order histogram is then defined as follows:

$$p\left( i \right)=\frac{P\left( i \right)}{\sum_{i=1}^{N_{g}} P\left( i \right)}$$

Let $\boldsymbol{V}_{ROI}$ and $\boldsymbol{V}$ denote the intensity values of all voxels within $V(x,y,z)$ with $N$ voxels and the whole image, respectively. The mean and center gray values within $V(x,y,z)$ are $\overline{V}$ and $\boldsymbol{C}$, respectively.

| **Number of voxels** | $\left\vert\boldsymbol{V}_{ROI} \right\vert$ | **Sum of intensities** | $\sum_{i}^{N} \boldsymbol{V}_{ROI}(i)$ |
| --- | --- | --- | --- |
| **Range** | $\text{max}\left( \boldsymbol{V}_{ROI} \right)-\text{min}\left( \boldsymbol{V}_{ROI} \right)$ | **Energy** | $N^{2}\sum_{i}^{N_{g}} {p\left( i \right)}^{2}$ |
| **Covered image intensity range** | $\frac{\max\boldsymbol{V}_{ROI}-\min\boldsymbol{V}_{ROI}}{\left( \max\boldsymbol{V}-\min\boldsymbol{V}+eps \right)}$ | **Entropy** | $-\sum_{i}^{N_{g}} \left[ p\left( i \right)*\log_{2} \left( p\left( i \right)+eps \right) \right]$ |
| **Maximum intensity value** | $\max\left( \boldsymbol{V}_{ROI} \right)$ | **Kurtosis** | $\frac{\sum_{i}^{N_{g}} \left[ p\left( i \right)*\left( \boldsymbol{C}\left( i \right)- \overline{V} \right)^{4} \right]}{\left( \sum_{i}^{N_{g}} \left[ p\left( i \right)*\left( \boldsymbol{C}\left( i \right)-\overline{V} \right) \right]+eps \right)^{2}}$ |
| **Mean intensity value** | $\frac{1}{N}\sum_{i}^{N} \boldsymbol{V}_{ROI}(i)$ | **Skewness** | $\frac{\sum_{i}^{N_{g}} \left[ p\left( i \right)*\left( \boldsymbol{C}\left( i \right)- \overline{V} \right)^{3} \right]}{\left( \sum_{i}^{N_{g}} \left[ p\left( i \right)*\left( \boldsymbol{C}\left( i \right)- \overline{V} \right)^{3} \right]+eps \right)^{\frac{3}{2}}}$ |
| **Median intensity value** | $\mathrm{med} \left( \boldsymbol{V}_{ROI} \right)$ | **Root means square** | $\sum_{i}^{N_{g}} \left[ p\left( i \right)*\boldsymbol{C}\left( i \right)^{2} \right]$ |
| **Minimum intensity value** | $\min\left( \boldsymbol{V}_{ROI} \right)$ | **Variance** | $\frac{1}{N-1}\sum_{i}^{N} \left( \boldsymbol{V}_{ROI}\left( i \right)- \overline{V} \right)^{2}$ |
| **Mean absolute deviation** | $\sum_{i}^{N_{g}} \left[ p\left( i \right)*(\boldsymbol{C}\left( i \right)- \overline{V}) \right]$ | **Standard deviation** | $\sqrt{\frac{1}{N-1}\sum_{i}^{N} \left( \boldsymbol{V}_{ROI}\left( i \right)- \overline{V} \right)^{2}}$ |
| **Uniformity** | $\sum_{i}^{N_{g}} p\left( i \right)^{2}$ |  |  |

1. Texture features

Although first-order features provide information on the gray-level distribution of the volume of interest, they do not describe information related to the relative positions of the various gray levels of the volume of interest. The methods most often used for texture analysis are the gray level co-occurrence matrix (GLCM) and the gray level run length matrix (GLRLM). In the use of GLCM, various textural features are extracted, and GLRLM characterizes coarse textures as having many pixels in a constant gray level run and fine textures as having few pixels in such a run. Both GLCM and GLRLM are matrix-based features as well as being constructed from 3-dimensional (3D) analysis of a volume of interest with 26-voxel connectivity, which are considered neighbors in all 13 directions in 3D.

- 1. GLCM features

Let $G$ denote the GLCM of a quantized volume $V(x,y,z)$ with isotropic voxel size and let $G_{\alpha,\delta}(i,j)$ represent the number of times that voxels of gray level *i* were neighbors with voxels of gray level $j$ in $V(x,y,z)$ in one of 13 directions of $\alpha$ and at a distance $\delta=1,2,3$. GLCM is the size of $N_{g}\times N_{g}$ where $N_{g}$ describes a pre-defined number of quantized gray level sets in $V(x,y,z)$. For each direction $\alpha$ and distance $\delta$, the normalized GLCM is obtained as follows:

$g_{\alpha,\delta}\left( i,j \right)=g\left( i,j \right)=\frac{G\left( i,j \right)}{\sum_{i=1}^{N_{g}} \sum_{j=1}^{N_{g}} G\left( i,j \right)}$.

Frequently used feature quantities for each direction and distance are also defined as follows:

- $\mu$ is the mean of $g\left( i,j \right)$
- $\sigma$ is the standard deviation of $g\left( i,j \right)$
- $g_{x}\left( i \right)$ is the marginal row probability of $g\left( i,j \right)$: $g_{x}\left( i \right)=\sum_{j}^{N_{g}} g\left( i,j \right)$,
- $\mu_{x}$ is the mean of $g_{x}\left( i \right)$
- $\sigma_{x}$ is the standard deviation of $g_{x}\left( i \right)$

| Mean and standard deviation of the followings for 13 directions and 3 distances | | | |
| --- | --- | --- | --- |
| **autocorrelation** | $\sum_{i}^{N_{g}} \sum_{j}^{N_{g}} i*j*g(i,j)$ | **Haralick correlation** | $\frac{1}{\sigma_{x}}\sum_{i}^{N_{g}} \sum_{j}^{N_{g}} \left( i*j*g\left( i,j \right) \right)-\mu_{x}$ |
| **cluster prominence** | $\sum_{i}^{N_{g}} \sum_{j}^{N_{g}} \left( i+j-2\mu\right)^{4}*g\left( i,j \right)$ | **inverse difference** | $\sum_{i}^{N_{g}} \sum_{j}^{N_{g}} \frac{g\left( i,j \right)}{1+\left\vert i-j \right\vert}$ |
| **cluster shade** | $\sum_{i}^{N_{g}} \sum_{j}^{N_{g}} \left( i+j-2\mu\right)^{3}*g\left( i,j \right)$ | **inverse difference normalized** | $\frac{1}{N_{g}}\sum_{i}^{N_{g}} \sum_{j}^{N_{g}} \frac{g\left( i,j \right)}{1+\left\vert i-j \right\vert}$ |
| **cluster tendency** | $\sum_{i}^{N_{g}} \sum_{j}^{N_{g}} \left( i+j-2\mu\right)^{2}*g\left( i,j \right)$ | **inverse difference moment** | $\sum_{i}^{N_{g}} \sum_{j}^{N_{g}} \frac{g\left( i,j \right)}{1+\left( i-j \right)^{2}}$ |
| **contrast** | $\sum_{i}^{N_{g}} \sum_{j}^{N_{g}} \left( i-j \right)^{2}*g\left( i,j \right)$ | **inverse difference moment normalized** | $\frac{1}{{N_{g}}^{2}}\sum_{i}^{N_{g}} \sum_{j}^{N_{g}} \frac{g\left( i,j \right)}{1+\left( i-j \right)^{2}}$ |
| **correlation** | $\frac{1}{\sigma}\sum_{i}^{N_{g}} \sum_{j}^{N_{g}} \left( i-\mu\right)\left( j-\mu\right)*g\left( i,j \right)$ | **inverse variance** | $\sum_{i}^{N_{g}} \sum_{j}^{N_{g}} \frac{g\left( i,j \right)}{\left( i-j \right)^{2}}$ |
| **difference average** | $\sum_{k}^{N_{g}} k*g_{x-y}\left( k \right)$ | **maximum probability** | $max\left( g\left( i,j \right) \right)$ |
| **difference entropy** | $-\sum_{k}^{N_{g}} g_{x-y}\left( k \right)*{log}_{2}\left( g_{x-y}\left( k \right)+eps \right)$ | **sum average** | $\sum_{k}^{2N_{g}} i*g_{x+y}\left( k \right)$ |
| **difference variance** | $\sum_{k}^{N_{g}} \left( k-\bar{g_{x-y}} \right)^{2}*g_{x-y}\left( k \right)$ | **sum entropy** | $-\sum_{k}^{2N_{g}} g_{x+y}\left( k \right)*{log}_{2}\left( g_{x+y}\left( k \right)+eps \right)$ |
| **dissimilarity** | $\sum_{i}^{N_{g}} \sum_{j}^{N_{g}} \left\vert i-j \right\vert*g\left( i,j \right)$ | **sum variance** | $\sum_{k}^{2N_{g}} \left( k-\bar{g_{x+y}} \right)^{2}*g_{x+y}\left( k \right)$ |
| **energy** | $\sum_{i}^{N_{g}} \sum_{j}^{N_{g}} {g\left( i,j \right)}^{2}$ | **variance** | $\sum_{i}^{N_{g}} \sum_{j}^{N_{g}} \left( i-\mu\right)^{2}g\left( i,j \right)$ |
| **entropy** | $-\sum_{i}^{N_{g}} \sum_{j}^{N_{g}} g\left( i,j \right)*{log}_{2}\left( g\left( i,j \right)+eps \right)$ |  |  |

- 1. GLRLM features

Let $Q$ denote the GLRLM of a quantized volume $V(x,y,z)$ with isotropic voxel size and $Q(i,j)$ represent the number of runs of gray level *i* with $j$ consecutive voxels in 1 of the 13 directions of $\alpha$. GLRLM is the size of $N_{g}\times N_{l}$ where $N_{g}$ describes the pre-defined number and $N_{l}$ represents the length of the longest run of quantized gray level sets in $V(x,y,z)$. $N_{p}$ is the number of voxels in $V(x,y,z)$.

| Mean and standard deviation of the followings for 13 directions and 3 distances | | | |
| --- | --- | --- | --- |
| **number of runs** | $N_{run}$ | **low gray level run emphasis** | $\frac{1}{N_{run}}\sum_{i}^{N_{g}} \sum_{j}^{N_{l}} \frac{1}{i^{2}}*Q\left( i,j \right)$ |
| **gray level nonuniformity** | $\frac{1}{N_{run}}\sum_{i}^{N_{g}} \left( \sum_{j}^{N_{l}} Q\left( i,j \right) \right)^{2}$ | **run length nonuniformity** | $\frac{1}{N_{run}}\sum_{j}^{N_{l}} \left( \sum_{i}^{N_{g}} Q\left( i,j \right) \right)^{2}$ |
| **high gray level run emphasis** | $\frac{1}{N_{run}}\sum_{i}^{N_{g}} \sum_{j}^{N_{l}} i^{2}*Q\left( i,j \right)$ | **run percentage** | $\frac{N_{run}}{N_{p}}$ |
| **long run emphasis** | $\frac{1}{N_{run}}\sum_{i}^{N_{g}} \sum_{j}^{N_{l}} j^{2}*Q\left( i,j \right)$ | **short run emphasis** | $\frac{1}{N_{run}}\sum_{i}^{N_{g}} \sum_{j}^{N_{l}} \frac{1}{j^{2}}*Q\left( i,j \right)$ |
| **long run high gray level emphasis** | $\frac{1}{N_{run}}\sum_{i}^{N_{g}} \sum_{j}^{N_{l}} {i^{2}*j}^{2}*Q\left( i,j \right)$ | **short run high gray level emphasis** | $\frac{1}{N_{run}}\sum_{i}^{N_{g}} \sum_{j}^{N_{l}} \frac{i^{2}}{j^{2}}*Q\left( i,j \right)$ |
| **long run low gray level emphasis** | $\frac{1}{N_{run}}\sum_{i}^{N_{g}} \sum_{j}^{N_{l}} \frac{j^{2}}{i^{2}}*Q\left( i,j \right)$ | **short run low gray level emphasis** | $\frac{1}{N_{run}}\sum_{i}^{N_{g}} \sum_{j}^{N_{l}} \frac{1}{i^{2}*j^{2}}*Q\left( i,j \right)$ |

**S2. Feature selection and classification methods in machine learning**

1. Feature selection methods
2. mRMR, minimum redundancy maximum relevance (1)

- mRMR evaluates features using relevancy–redundancy trade-offs and removes redundant features by considering pairwise feature correlation measured by mutual information. The selected features are mutually far away from each other and have high correlation in their classification variables.

1. CFS, correlation based feature selection (2)

- CFS quickly identifies and screens irrelevant, redundant, and noisy features and identifies relevant features as long as their relevance does not strongly depend on other features. In previous theses, CFS gave comparable results to the wrapper-type feature selector, and even outperformed the wrapper type on small datasets. CFS can execute processing many times faster than the wrapper type, which allows it to scale to larger datasets.

1. RFE, recursive feature elimination (3)

- RFE selects features by recursively considering smaller and smaller sets of features. The estimator is trained on an initial set of features and weights are assigned to each. Then the features whose absolute weights are the smallest are eliminated from the current features in a backwards elimination manner. This procedure is recursively repeated until the desired number of features is reached.

1. Classification methods
2. RBF-SVM, radial basis function SVM (4)

- RBF-SVM is a nonlinear version of linear SVM with Gaussian kernel function that projects original features onto a higher dimensional space via a nonlinear mapping function where it becomes linearly separable. The box constraint is heuristically obtained in the log-scaled range (1e-3, 1e3) and the Gaussian kernel function is automatically scaled with a heuristic procedure provided by MATLAB.

1. Boosted generalized linear mixed model (5)

- Generalized linear models (GLMM) are an extension of generalized linear models that incorporate random effects. As a consequence of heavy computational problems in GLMM modeling, it has been restricted to a few predictor variables. Boosting was developed as a method to improve classification. Algorithms in boosted GLMM allow for estimation of generalized mixed models with high dimensional predictor structures. The algorithm is constructed as a component-wise boosting method and performs variable selection with the complexity of the resulting estimator being determined by information criteria.

1. RF, random forest

- RF provides an improvement to bagging with a modification step of random sampling of predictors. R package (version 3.2.4, R Core Team, Vienna, Austria) “randomForest” with caret interface was used for the implementation. The parameter “ntree” was set to 500 and “mtry” was varied with values 2:3:29 (2 to 29 with an increment step of 3)
- RF is an extension of decision tree(DT) that includes more DT classifiers. This algorithm was built by a combination of random selection of observations and a random selection of a subset of attributes; 101 decision trees were used to predict classes of new observations.

**References for S1.**

1. Auffarth B, López M, Cerquides J. Comparison of Redundancy and Relevance Measures for Feature Selection in Tissue Classification of CT Images. Advances in Data Mining.248.

2. Hall MA. Correlation-based feature selection for machine learning: The University of Waikato; 1999.

3. Guyon I, Weston J, Barnhill S, Vapnik V. Gene selection for cancer classification using support vector machines. Machine learning. 2002;46(1):389-422.

4. Schölkopf B, Smola AJ. Learning with kernels: support vector machines, regularization, optimization, and beyond (adaptive computation and machine learning). The MIT Press Cambridge; 2001.

5. Tutz G, Groll A. Generalized linear mixed models based on boosting. Statistical Modelling and Regression Structures: Springer; 2010. p. 197-215.

**S3. Results of metric 1 in each magnetic resonance (MR) imaging dataset**

| Feature selection method | Classification method | Mean AUCs (10-fold cross-validation) |
| --- | --- | --- |
| CE-T1WI |  |  |
| mRMR | Radial-basis SVM | 0.834 |
|  | GLM-boosting | 0.921 |
|  | Regularized RF | 0.893 |
| Correlation-based FS | Radial-basis SVM | 0.924 |
|  | GLM-boosting | 0.909 |
|  | Regularized RF | 0.901 |
| Backward elimination | Radial-basis SVM | 0.852 |
|  | GLM-boosting | 0.918 |
|  | Regularized RF | 0.869 |
| ADC |  |  |
| mRMR | Radial-basis SVM | 0.827 |
|  | GLM-boosting | 0.894 |
|  | Regularized RF | 0.885 |
| Correlation-based FS | Radial-basis SVM | 0.868 |
|  | GLM-boosting | 0.894 |
|  | Regularized RF | 0.910 |
| Backward elimination | Radial-basis SVM | 0.656 |
|  | GLM-boosting | 0.852 |
|  | Regularized RF | 0.836 |
| CE-T1WI+ADC |  |  |
| mRMR | Radial-basis SVM | 0.917 |
|  | GLM-boosting | 0.926 |
|  | Regularized RF | 0.927 |
| Correlation-based FS | Radial-basis SVM | 0.933 |
|  | GLM-boosting | 0.918 |
|  | Regularized RF | 0.886 |
| Backward elimination | Radial-basis SVM | 0.617 |
|  | GLM-boosting | 0.943 |
|  | Regularized RF | 0.877 |

Abbreviations: CE-T1WI = contrast-enhanced T1-weighted image; ADC = apparent diffusion coefficient; mRMR = minimum redundancy maximum relevance; FS= feature selection; SVM = support vector machine; GLM= generalized linear mixed model; RF = random forest

**S4. Selected Features from the Best Combination of Feature Selection and Classifier in Metric 1 according to Imaging Sequences**

| Radiomic feature | Feature type | | Wavelet transformation |
| --- | --- | --- | --- |
| CE-T1WI | Correlation-based FS with Radial-basis SVM | | |
| Inverse variance: std | GLCM(3) | Original | |
| Difference entropy: std | GLCM(1) | Original | |
| Inverse Variance: mean | GLCM(2) | Original | |
| Inverse difference (Homogeneity 1) : mean | GLCM(2) | Original | |
| Short run high gray-level emphasis (SRHGLE): std | GLRLM | HHL | |
| Short run emphasis (SRE): mean | GLRLM | Original | |
| Haralick Correlation: std | GLCM(3) | HHL | |
| Compactness 1 | shape/volume | Original | |
| Difference variance: std | GLCM(1) | Original | |
| ADC | Correlation-based FS with Regularized RF | | |
| Cluster shade: std | GLCM (2) | Original | |
| Maximum probability: std | GLCM (2) | LLL | |
| Inverse Variance: std | GLCM (3) | HHH | |
| Sphericity | shape/volume | LLL | |
| Cluster Shade: mean | GLCM (1) | HLL | |
| Sphericity | Minimum | Original | |
| High gray-level run emphasis (HGLRE): std | GLRLM | HHL | |
| Skewness | first order | LLL | |
| Run percentage (RP): mean |  |  | |
| CE-T1WI+ADC | Backward elimination with GLM-boosting | | |
| ADC) Cluster shade: std | GLCM (2) | Original | |
| CE-T1WI) Short run high gray-level emphasis (SRHGLE): std | GLRLM | HHL | |
| CE-T1WI) Inverse difference (Homogeneity 1) : mean | GLCM(2) | Original | |
| CE-T1WI) Difference entropy: std | GLCM(1) | Original | |
| CE-T1WI) Inverse Variance: std | GLCM (3) | HHH | |
| ADC) Sphericity | shape/volume | LLL | |
| CE-T1WI) High gray-level run emphasis (HGLRE): std | GLRLM | HHL | |
| ADC) Maximum probability: std | GLCM (2) | LLL | |
| CE-T1WI) Difference entropy: std | GLCM(1) | Original | |
| ADC) Inverse Variance: mean | GLCM(2) | Original | |

Abbreviations: CE-T1WI = Contrast-enhanced T1-weighted imaging, std = standard deviation, GLCM= gray level co-occurrence matrix, GLRLM = gray-level run-length matrix, H= high-pass filter, L= low pass filter

**S5. Results of adding deep features to radiomics features in the random forest classifier**

| Internal validation |  | AUC | Accuracy | Sensitivity | Specificity |
| --- | --- | --- | --- | --- | --- |
| Radiomic features | CE-T1W1 | 0.9722 | 90.00 | 83.33 | 94.44 |
|  | ADC | 0.8981 | 76.67 | 58.33 | 88.89 |
|  | CE-T1W1 + ADC | 0.9907 | 93.33 | 83.33 | 100 |
| Radiomic features +  CNN-based deep features | CE-T1W1 | 0.4676 | 90.00 | 83.33 | 94.44 |
|  | ADC | 0.5579 | 83.33 | 75.00 | 88.89 |
|  | CE-T1W1 + ADC | 0.4722 | 83.33 | 75.00 | 88.89 |
| External validation |  |  |  |  |  |
| Radiomic features | CE-T1W1 | 0.8903 | 83.33 | 78.57 | 85.71 |
|  | ADC | 0.8852 | 78.57 | 85.71 | 75.00 |
|  | CE-T1W1 + ADC | 0.9260 | 85.71 | 85.71 | 85.71 |
| Radiomic features +  CNN-based deep features | CE-T1W1 | 0.3878 | 83.33 | 78.57 | 85.71 |
|  | ADC | 0.8291 | 73.81 | 85.71 | 67.86 |
|  | CE-T1W1 + ADC | 0.7895 | 83.33 | 78.57 | 85.71 |
